# Supplementary material for: Players’ strategy selection in co-governance and supervision of internet platforms’ monopolistic behaviors: A study on new media participation
Source: PLoS One. 2024 Apr 16;19(4):e0299076. doi: 10.1371/journal.pone.0299076 (PMC11020868; doi:10.1371/journal.pone.0299076)
Supplement: S1 File — (DOCX) [file pone.0299076.s001.docx]

1. There is no data set, all the data is in the manuscript.

2．The sixth part of the article，these following data are simulation data and are used for simulation analysis

**Simulation Analysis**

To reflect the impact of changes in decision variables on players’ decision-making more intuitively, a numerical simulation, using MATLAB 2020b, was conducted to verify the effectiveness of the stability analysis.

We set the cost of the government applying traditional supervision $C_{gl}=3$, the cost of co-governance and supervision $C_{gh}=7.5$, the information value obtained from the co-governance platform $V_{\text{g}}=4.0$, the penalty imposed on monopoly $F_{e}=3.0$, the increased public trust $H_{g}=1$, and the negative impact of not being able to identify monopolistic behavior $D_{g}=1.5$. The income generated by Internet platforms’ when operating in compliance with rules and regulations $R_{el}=3$, the income when operating as a monopoly $R_{eh}=4$, the cost of operating in compliance with rules and regulations $C_{eh}=2$, the cost of operating as a monopoly $C_{el}=1$. The cost of new media participating in co-governance $C_{\text{m}}$ = 3, the information value obtained by participating in co-governance $V_{m}$ = 2, the reward given to the public for making complaints/reports $R_{p}$ = 1. The reward given by the new media to the public $R_{P}=1.0$, the cost of making a complaint/report $C_{p}=2.5$. Internet platforms’ loss of reputation when operating as a monopoly and being exposed by new media $N_{e}=2$, whilst the increased reputation generated through positive exposure on new media $I_{e}=1$, and the loss of the rights and interests to the public by operating as a monopoly $L_{p}=1$. Government’s ability to identify monopolistic behavior when applying traditional supervision $\alpha$ was 0.3, while the increase in identification ability when applying co-governance and supervision $\Delta\alpha$ was 0.4.

Import the following code into MATLAB 2020b to get the following Figure 6. Influence of the Probability of New Media Participation

Z=0.2

dA=inline('[y(1)*(1-y(1))*(-4.5+(1-y(2))*5.6);y(2)*(1-y(2))*(-0.78+1.2*y(1)+0.16*y(1));y(3)*(1-y(3))*(-2.5+0.2*(1-y(2))*1)]','t','y');

tspan=[0 100];

y0=[0.6,0.2,0.5];

[t,y]=ode45(dA,tspan,y0);

figure,plot(t,y(:,1),'k--');

hold on

plot(t,y(:,2),'k*');

hold on

plot(t,y(:,3),'k+');

Z=0.5

dA=inline('[y(1)*(1-y(1))*(-4.5+(1-y(2))*5.6);y(2)*(1-y(2))*(-0.6+1.2*y(1)+0.3+0.4*y(1)); y(3)*(1-y(3))*(-2.5+0.5* (1-y(2))*1)]','t','y');

tspan=[0 150];

y0=[0.6,0.2,0.5];

[t,y]=ode45(dA,tspan,y0);

figure,plot(t,y(:,1),'k--');

hold on

plot(t,y(:,2),'k*');

hold on

plot(t,y(:,3),'k+');

Z=0.8

dA=inline('[y(1)*(1-y(1))*(-4.5+(1-y(2))*5.6);y(2)*(1-y(2))*(0.18+1.2*y(1)+0.64*y(1));y(3)*(1-y(3))*(-2.5+0.8*(1-y(2))*1)]','t','y');

tspan=[0 100];

y0=[0.6,0.2,0.5];

[t,y]=ode45(dA,tspan,y0);

figure,plot(t,y(:,1),'k--');

hold on

plot(t,y(:,2),'k*');

hold on

plot(t,y(:,3),'k+');


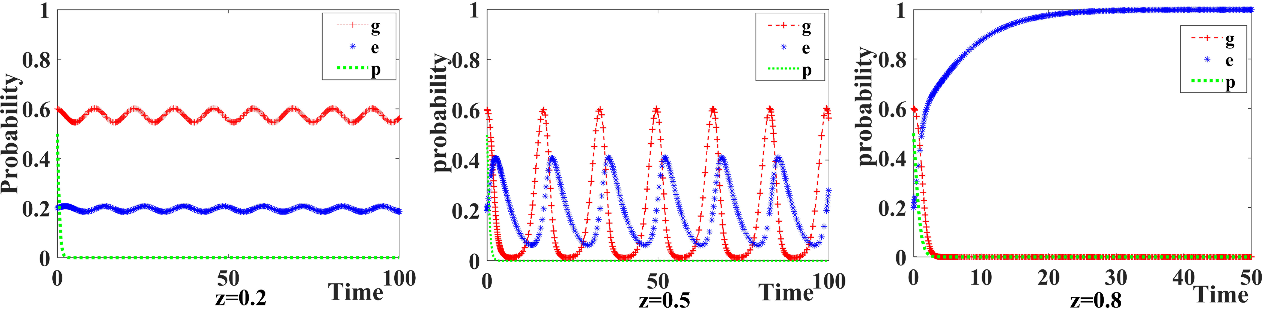


**Figure 6. Influence of the Probability of New Media Participation**

Import the following code into MATLAB 2020b to get the following Figure 7. Influence of New Media Participation on Reputation Value

$$I_{e}=0.0,N_{e}=0.0$$

dA=inline('[y(1)*(1-y(1))*(-4.5+(1-y(2))*5.6);y(2)*(1-y(2))*(-1.1+1.2*y(1));y(3)*(1-y(3))*(-3+y(4)*(1-y(2))*1); y(4)*(1-y(4))*(-0.5+y(3)*(1-y(2))*1)]','t','y');

tspan=[0 200];

y0=[0.6,0.4,0.8,0.5];

[t,y]=ode45(dA,tspan,y0);

figure,plot(t,y(:,1),'k--');

hold on

plot(t,y(:,2),'k*');

hold on

plot(t,y(:,3),'k-.');

hold on

plot(t,y(:,4),'k+')

$$I_{e}=2.0,N_{e}=2.0$$

dA=inline('[y(1)*(1-y(1))*(-4.5+(1-y(2))*5.6);y(2)*(1-y(2))*(-2+y(3)*2+0.3*3+1.2*y(1)+0.6*y(3)+0.8*y(1)*y(3));y(3)*(1-y(3))*(-3+y(4)*(1-y(2))*1);y(4)*(1-y(4))*(-2.5+y(3)*(1-y(2))*1)]','t','y');

tspan=[0 200];

y0=[0.6,0.3,0.3,0.3];

[t,y]=ode45(dA,tspan,y0);

figure,plot(t,y(:,1),'k--');

hold on

plot(t,y(:,2),'k*');

hold on

plot(t,y(:,3),'k-.');

hold on

$$I_{e}=4.0,N_{e}=4.0$$

dA=inline('[y(1)*(1-y(1))*(-4.5+(1-y(2))*5.6); y(2)*(1-y(2))*(-1.1+y(3)*4+1.2*y(1)+2.7*y(3)+3.6*y(1)*y(3));y(3)*(1-y(3))*(-3+y(4)*(1-y(2))*1);y(4)*(1-y(4))*(-0.5+y(3)*(1-y(2))*1)]','t','y');

tspan=[0 100];

y0=[0.6,0.4,0.8,0.5];

[t,y]=ode45(dA,tspan,y0);

figure,plot(t,y(:,1),'k--');

hold on

plot(t,y(:,2),'k*');

hold on

plot(t,y(:,3),'k-.');

hold on

plot(t,y(:,4),'k+')


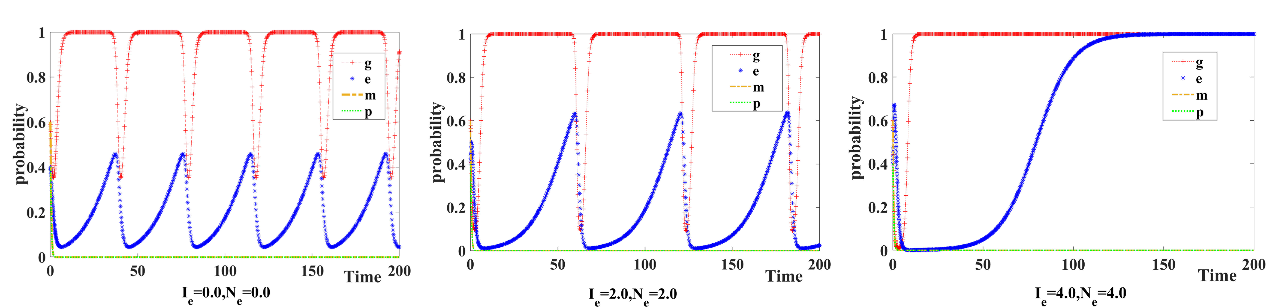


**Figure 7. Influence of New Media Participation on Reputation Value**

Import the following code into MATLAB 2020b to get the following Figure 8. Influence of Additional Costs

$$\Delta C_{g}=0.0,\Delta C_{e}=0.0,\Delta C_{m}=0.0$$

function dxdt=differential4(t,x)

dxdt=[x(1)*(1-x(1))*(5.6*(1-x(2)));x(2)*(1-x(2))*(-0.1+1.6*x(3)+1.2*x(1)+x(3)*x(1)*0.8);x(3)*(1-x(3))*(-1+0.5*(1-x(2))*1)];

end

for i=0.001:0.1:1

for j=0.001:0.1:1

for l=0.001:0.1:1

[T,Y]=ode45('differential4',[0 25],[i j l]);

figure(1)

grid on

plot3(Y(:,1),Y(:,2),Y(:,3));

hold on

end

end

end

$$\Delta C_{g}=4.0,\Delta C_{e}=1.0,\Delta C_{m}=3.0$$

function dxdt=differential4(t,x)

dxdt=[x(1)*(1-x(1))*(-4+5.6*(1-x(2)));x(2)*(1-x(2))*(-1.1+1.6*x(3)+1.2*x(1)+x(3)*x(1)*0.8);x(3)*(1-x(3))*(-3+0.5*(1-x(2))*1)];

end

for i=0.001:0.1:1

for j=0.001:0.1:1

for l=0.001:0.1:1

[T,Y]=ode45('differential4',[0 25],[i j l]);

figure(1)

grid on

plot3(Y(:,1),Y(:,2),Y(:,3));

hold on

end

end

end

$$\Delta C_{g}=8.0,\Delta C_{e}=5.0,\Delta C_{m}=6.0$$

function dxdt=differential4(t,x)

dxdt=[x(1)*(1-x(1))*(-8+5.6*(1-x(2)));x(2)*(1-x(2))*(-5.1+1.6*x(3)+1.2*x(1)+x(3)*x(1)*0.8);x(3)*(1-x(3))*(-6+0.5*(1-x(2))*1)];

end

for i=0.001:0.1:1

for j=0.001:0.1:1

for l=0.001:0.1:1

[T,Y]=ode45('differential4',[0 25],[i j l]);

figure(1)

grid on

plot3(Y(:,1),Y(:,2),Y(:,3));

hold on

end

end

end

**
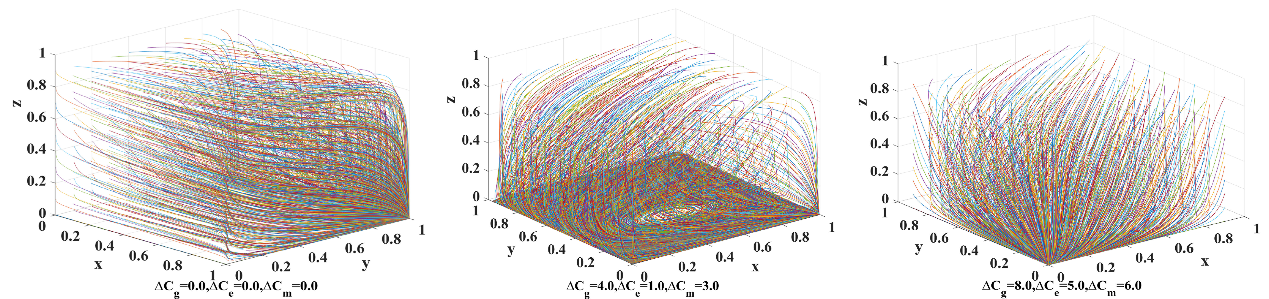
**

**Figure 8. Influence of Additional Costs**
