# Supplementary material for: Players’ strategy selection in co-governance and supervision of internet platforms’ monopolistic behaviors: A study on new media participation
Source: PLoS One. 2024 Apr 16;19(4):e0299076. doi: 10.1371/journal.pone.0299076 (PMC11020868; doi:10.1371/journal.pone.0299076)
Supplement: Attachment — Submitted filename: Response to Reviewers-23-39623.docx [file pone.0299076.s002.docx]

**Responses to Editor and Reviewers**

Dear Editor,

Thank you very much for your critical reading and valuable comments about our paper submitted to *PLOS ONE* (Manuscript ID: ***PONE-D-23-39623***). The manuscript’s title is “*Players’ Strategy Selection in Co-Governance and Supervision of Internet Platforms’ Monopolistic Behaviors: A Study on New Media Participation*”.

Those comments were very helpful for providing direction for our further studies. We have tried our best to revise our manuscript according to the comments. Attached, please find the *PONE-D-23-39623 Revised Manuscript*, which we would like to resubmit for your kind consideration. The following is a detailed explanation how we have complied with the reviewers’ suggestions.

**Responds to the reviewers’ comments:**

**Reviewer 1**

**Comment #1:**

(1) The cases in the introduction need to indicate the source, increase the authenticity and rigor of the event.

Response: Thank you very much for pointing out the problem. We have indicated the source of the cases in the introduction to increase the authenticity and rigor of the event. such as, In response to antitrust incidents involving Internet platforms, the United States has tightened previously relaxed policies on the platform economy (www.justice.com).

China has also focused on improving regulatory systems and mechanisms, innovating supervision concepts and methods, and developing highly knowledgeable and professional teams to enhance its anti-monopoly regulatory capabilities(www.gov.cn).
We have marked the modification in red in *PONE-D-23-39623 Revised Manuscript.*

**Comment #2:**

(2) The revision of legal documents should be added as much as possible to indicate the authenticity of the event.

**Response**: Thank you very much for pointing out the problem. We have Add the date of enactment of the law. Such as "The Fair Online Platform Intermediary Transactions Act, drafted by the Korea Fair Trade Commission, requires Internet platforms to provide a written contract that specifies the terms directly related to sellers’ interests during intermediary transactions in March 2021. ","the Competition Commission, which conducts comprehensive anti-monopoly investigations on platform companies; and the Competition Appeal Court) in November 18, 2021. " We have marked the modification in red in *PONE-D-23-39623 Revised Manuscript.*

**Comment #3:**

(3) Before modeling, please explain the relevance of using evolutionary game to solve the antitrust problem of Internet platform enterprises.

**Response**: Thank you very much for the valuable suggestions. In order to increase the logic of the paper, at the beginning of Chapter 3, we explained the reasons for choosing this research method. "This paper chooses evolutionary game as the research method because it can explain the dynamic process of the evolution of each stakeholder's strategic choice and explain why this state has been reached and how to reach. Therefore, considering a conceptual model of the anti-monopoly co-governance and supervision system, involving the government, Internet platforms, new media, and the public, is presented in Figure 1." We also marked modification in red in *PONE-D-23-39623 Revised Manuscript.*

**Comment #4:**

(4) Equation (5) and Equation (12) pay attention to the range of values at the ends of [0, 1], which need to be modified, do not repeat the values.

**Response**: Thank you very much for your careful review work. We modify the value range at the end of [0,1] from [0,1] to (0,1). After the modification, the value range does not contain repeated values. We have marked the modification in red in *PONE-D-23-39623 Revised Manuscript.*

**Comment #5:**

(5) Whether the results of the model proof and the results of the simulation analysis can be further combined to make the explanation clearer.

**Response**: Thank you very much for the valuable suggestions. We have added analysis in the simulation analysis section. We have marked the modification in red in *PONE-D-23-39623 Revised Manuscript.*

**Comment #6:**

(6) Whether the conclusion part of the article can be combined with the reality of antitrust behavior to make more targeted suggestions.

**Response**: Thank you very much for the valuable suggestions. We have marked the modification in red in *PONE-D-23-39623 Revised Manuscript.*

**Reviewer 2**

**Comment #1:**

(1) Please the author to cite the latest literature in this field from the past three years 2021-2023.

**Response:** Thank you very much for the valuable suggestions. We have cited the latest literature in this field from the past three years 2021-2023.We have marked the modification in red in *PONE-D-23-39623 Revised Manuscript.*

**Comment #2:**

(2) The manuscript is 35 pages long, please compress it appropriately by the author.

**Response**: Thank you very much for the valuable suggestions. We have read through, revised, condensed and compressed the article many times. After re-reading and summarizing, the article has been compressed.

**Comment #3:**

(3) Please the author to improve the clarity of the Figures to achieve above 300*300 DPI.

**Response**: Thank you very much for the valuable suggestions. We have processed the picture according to your request and we have improved the clarity of the Figures to achieve above 300*300 DPI.

**Reviewer 3**

**Comment #1:**

(1) The relevant literature review of the previous part of the lack of important literature with game theory research as a theoretical support, should be added in the literature review of game theory research related literature.

**Response**: Thank you very much for the valuable suggestions. We have added in the literature review of game theory research related literature. Such as“Daniel Friedman. On economic applications of evolutionary game theory[J]Journal of Evolutionary Economics,1998(1)”“Alexander JMK. Evolutionary game theory[J].Elements in Decision Theory and Philosophy, 2023.”Owen G. Game theory[M]. Emerald Group Publishing, 2013.“We have marked the modification in red in *PONE-D-23-39623 Revised Manuscript.*

**Comment #2:**

(2) In the chapter of model assumption and construction, please explain why the method of evolutionary game is used to analyze and solve this problem.

**Response**: Thank you very much for the valuable suggestions. In order to increase the logic of the paper, at the beginning of Chapter 3, we explained the reasons for choosing this research method. "This paper chooses evolutionary game as the research method because it can explain the dynamic process of the evolution of each stakeholder's strategic choice and explain why this state has been reached and how to reach. Therefore, considering a conceptual model of the anti-monopoly co-governance and supervision system, involving the government, Internet platforms, new media, and the public, is presented in Figure 1." We also marked modification in red in *PONE-D-23-39623 Revised Manuscript.*

**Comment #3:**

(3) Whether the data selection in the simulation analysis can be realized by using the real data now?

**Response**: Thank you very much for your question. Here we use numerical simulation, combining the real situation and the researches of other scholars to set the values, and achieve the purpose of problem research through numerical calculation and image display. At present, it is impossible to obtain actual data ideally, which is also a problem that we need to work hard to overcome in the future.

**Comment #4:**

(4) It is suggested to read through the article again to improve and enhance the readability of the article.

**Response**: Thank you very much for the valuable suggestions. I have read through the article again to improve and enhance the readability of the article.

We have tried our best to improve the manuscript and made some changes according to the reviewers’ comments. We earnestly appreciate reviewers’ professional work and hope that the corrections will make our manuscript suitable for publication in *PLOS ONE*. We are looking forward to receiving comments from reviewers in the future.

Once again, thank you very much for your valuable comments and suggestions.

Best wishes.

Sincerely yours,

Tongshui Xia

13, Jan, 2024
